# Siderotic cataract with no signs of intraocular foreign body

**DOI:** 10.1186/s12886-017-0424-4

**Published:** 2017-03-14

**Authors:** Ke-Ke Zhang, Wen-Wen He, Yi Lu, Xiang-Jia Zhu

**Affiliations:** 1 Eye Institute, Eye and Ear, Nose, and Throat Hospital, Fudan University, Shanghai, 200031 China; 2 Department of Ophthalmology, Eye and Ear, Nose, and Throat Hospital, Fudan University, Shanghai, 200031 China; 3 Key Laboratory of Myopia, Ministry of Health PR China, Shanghai, 200031 China; 4 Shanghai Key Laboratory of Visual Impairment and Restoration, Fudan University, Shanghai, 200031 China

**Keywords:** Ocular siderosis, Cataract, Intraocular foreign body, Histopathology, Electroretinography, Case report

## Abstract

**Background:**

Ocular siderosis is a clinical condition induced by deposition of an iron-containing intraocular foreign body. We report a unique case of histopathologically proven lens siderosis in a young woman with a preceding history of trauma but no signs of retained intraocular foreign body.

**Case presentation:**

A 32-year-old woman presented with an opacified lens showing brownish deposits on the anterior capsule and underwent cataract surgery. Preoperative ophthalmic examination did not show any retained intraocular foreign body. Histopathologic staining of the anterior capsule confirmed the presence of iron deposits and macrophages. Electroretinography examination performed in the postoperative period showed the changes characteristic of retinal degeneration in ocular siderosis.

**Conclusion:**

This case illustrates the importance of close monitoring of patients with a history of trauma or previous penetrating injury to the eye, even if there is no intraocular foreign body, because they might develop ocular siderosis at a later stage. This case report underscores the importance of electroretinography and histopathologic analysis, in addition to ophthalmic examination, in the diagnosis of ocular siderosis.

**Electronic supplementary material:**

The online version of this article (doi:10.1186/s12886-017-0424-4) contains supplementary material, which is available to authorized users.

## Background

Ocular siderosis is a clinical condition caused by deposition of an iron-containing intraocular foreign body [1] and may occur 18 days to 8 years after ocular injury [2]. Clinical findings include iris heterochromia, pupillary mydriasis, iron deposition on the corneal endothelium and anterior lens capsule, cataract formation, lens subluxation, secondary glaucoma, uveitis, and retinal pigment changes or degeneration [3, 4]. Patients usually present with a history of ocular trauma, although some may remain asymptomatic and present only later when their visual acuity has decreased [2, 5]. We report a rare case of histopathologically proven lens siderosis in a young woman with a history of trauma but no signs of retained intraocular foreign body.

## Case presentation

A 32-year-old Chinese woman presented to the outpatient department on May 13, 2016 complaining of a 6-month history of progressive blurring of vision in her right eye. She has no past medical history of note, but gave an uncertain history of a foreign body made of iron hitting her right eye 1 year earlier. After being given eyedrops by a doctor at a local hospital, she did not seek further medical care until she noticed further worsening of her vision.

**Fig. 1:**
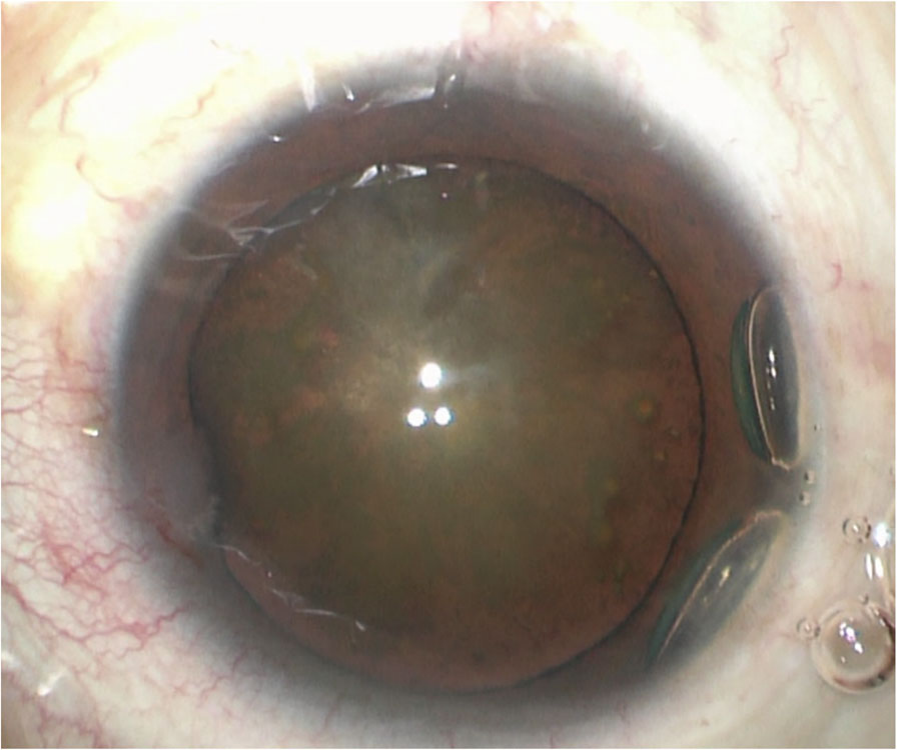
Anterior segment image of the right eye. This image was taken during cataract surgery and shows an opacified lens with brownish deposits on the anterior lens capsule

**Fig. 2:**
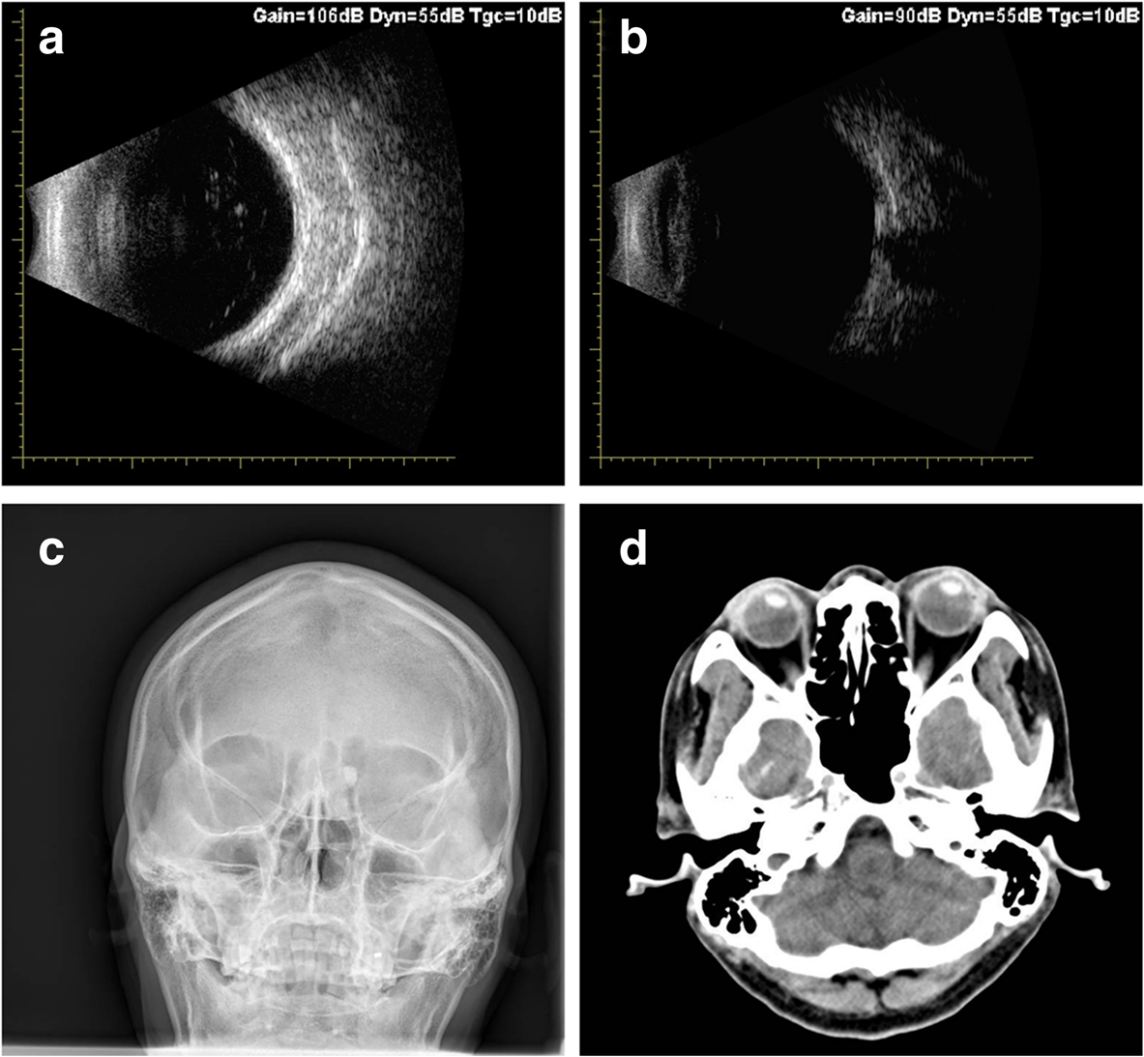
(**a**-**b**) Ultrasound B-scan of the posterior segment in low gain settings. The posterior capsule is intact and there is no foreign body seen intraocularly. (**c**) X-ray of orbits showing no radiopaque foreign body in Water's view. (**d**) Computed tomography scan showing normal intact globe with no intraocular foreign body in the right eye

The patient’s visual acuity was 6/20 in the right eye (OD) and 20/20 in the left eye (OS); intraocular pressures were normal (16.9 mmHg OD and 17.2 mmHg OS, by non-contact tonometry). Both pupils were round and there was no relative afferent pupillary defect. Examination of the anterior and posterior segments of the left eye was normal. However, in the right eye, slit-lamp biomicroscopy revealed a corneal macula inferiorly, indicating a possible perforating wound previously. Lens opacification was also observed in the right eye, with clumps of brownish pigment on the anterior lens capsule clinically suggestive of siderotic cataract (Fig. 1). There was no evidence of any breach in the anterior lens capsule. The anterior chamber was clear and of medium depth. As the cataract did not entirely obscured the fundus, it is possible for us to perform the detailed fundus examination with a three-mirror lens, which showed no retinal detachment or signs of foreign body. Using slit-lamp examination and gonioscopy, no intraocular foreign body was visualized in the anterior segment of the injured eye before and after mydriasis. Optical coherence tomography vaguely indicated no retinal detachment in the posterior pole region (Additional file 1: Figure S1). An ultrasound B-scan (10 MHz) showed moderately dense, mobile, vitreous opacities with posterior vitreous detachment and no retinal tear or detachment. No intralenticular foreign body was seen on low gain (Figs. 2a and b). An orbital X-ray (Fig. 2c) and a 3.0-mm thin-sliced computed tomography scan (Fig. 2d) did not show any retained intraocular foreign body.

**Fig. 3:**
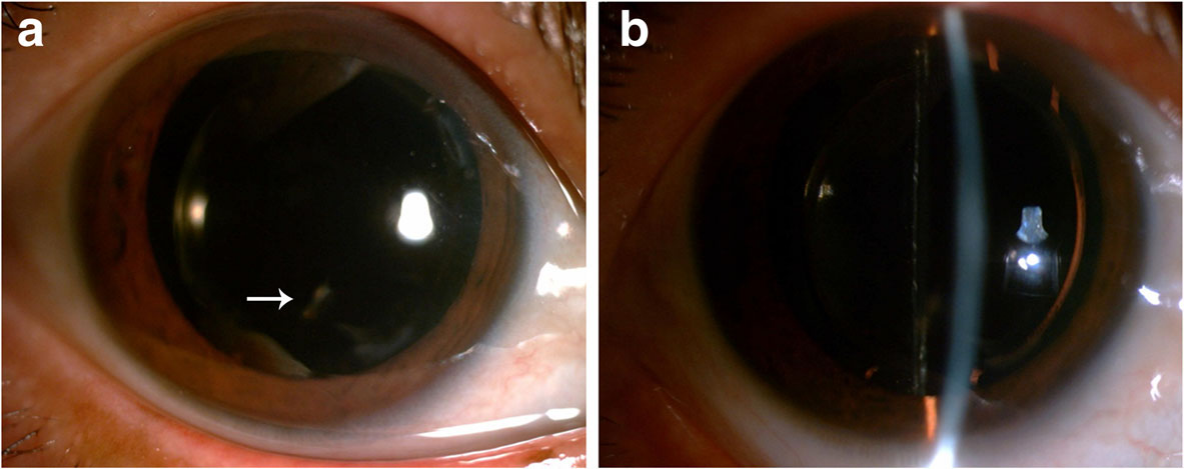
Postoperative anterior segment images of the right eye after mydriasis through direct diffuse illumination (**a**) and indirect illumination (**b**). Despite a corneal macula inferiorly (*arrow*), the right eye showed no signs of inflammation and had a clear anterior chamber of medium depth with a well-centered intraocular lens

On the basis of the above findings, the patient was diagnosed with siderotic cataract and underwent an uneventful cataract phacoemulsification with implantation of a posterior chamber intraocular lens (IOL) and capsular tension ring under regional anesthesia in her right eye on May 23, 2016. Dilated fundus examination was also performed intraoperatively to determine if there were any siderotic changes on the retina. However, no retained intraocular foreign body, retinal detachment, retinal tear, or vitreous hemorrhage was seen. The anterior lens capsule was obtained during continuous curvilinear capsulorhexis and sent for histopathology. The patient was treated with Cravit Eye Drops (Alcon Laboratories, Inc., Fort Worth, TX, USA) QID OD, Pred Forte Eye Drops (Allergan Pharmaceuticals, Inc., Dublin, Ireland) QID OD, and Diclofenac Sodium Eye Drops (Shenyang Xingqi Pharmaceutical Co. Ltd, Shenyang, China) QID OD. One week after the surgery, her best uncorrected visual acuity improved to 16/20, and her best corrected visual acuity improved to 20/20 with a corneal macula inferiorly, a clear anterior chamber of medium depth, and a well-centered IOL (Fig. 3).

**Fig 4:**
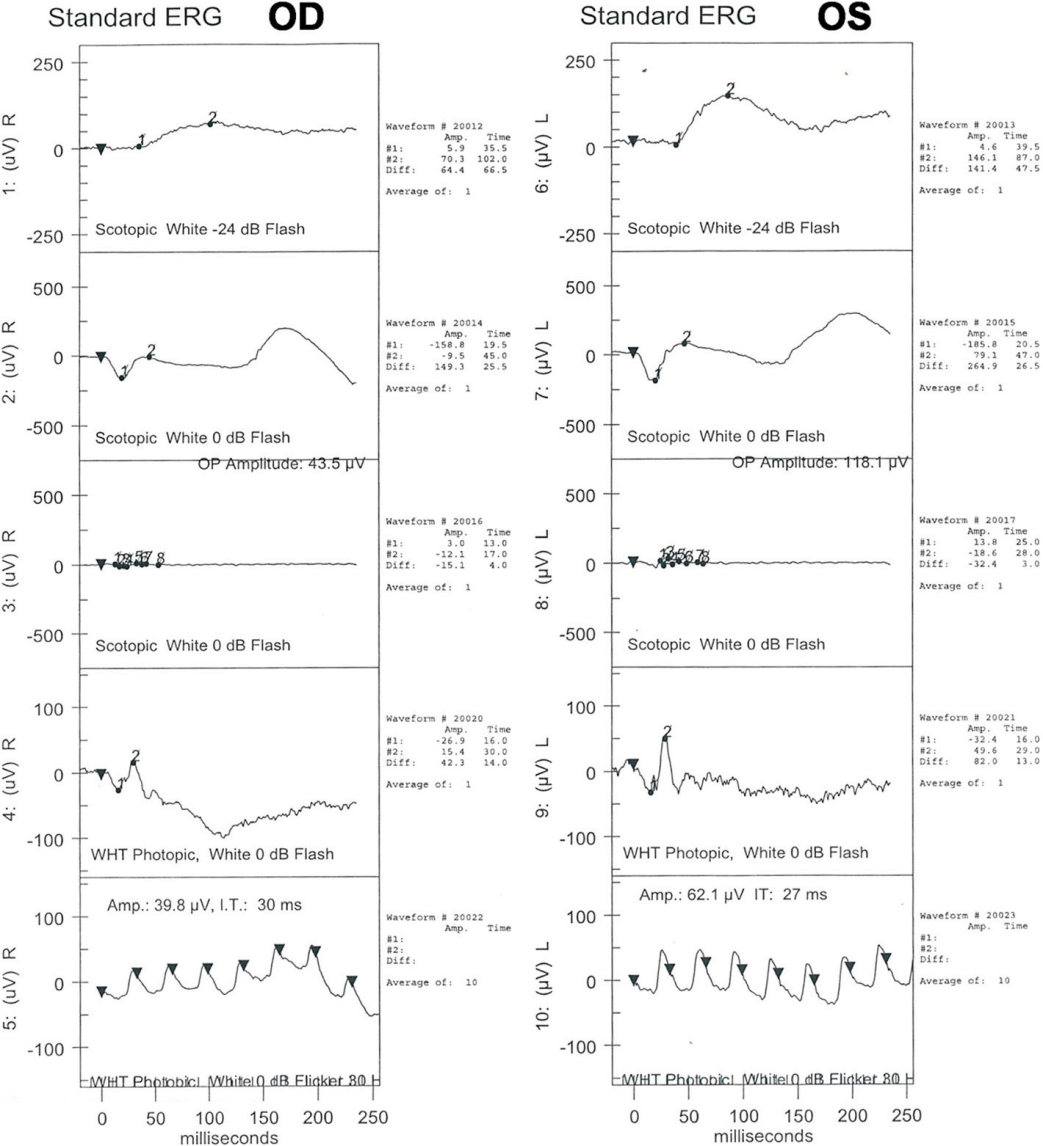
Electroretinography examination of both eyes performed in the postoperative period. Electroretinography was basically normal in the left eye (OS) for combined, scotopic, and photopic responses, as well as a normal flicker response. However, these responses were decreased in the right eye (OD), which demonstrated reduced A-wave and B-wave amplitudes compared with the normal left eye. (From top to bottom: rod, mixed cone-rod, OP, single-flash cone, and 30-Hz flicker ERG responses)

Electroretinography (ERG) examination performed in the postoperative period revealed the changes characteristic of retinal degeneration with reduced A-wave and B-wave amplitudes (Fig. 4). It also showed implicit time delayed and decreased B:A ratio in both rod and cone system. ERG was normal in the left eye for combined, scotopic, and photopic responses, as well as a normal flicker response. However, these responses were decreased in the right eye.

**Fig. 5:**
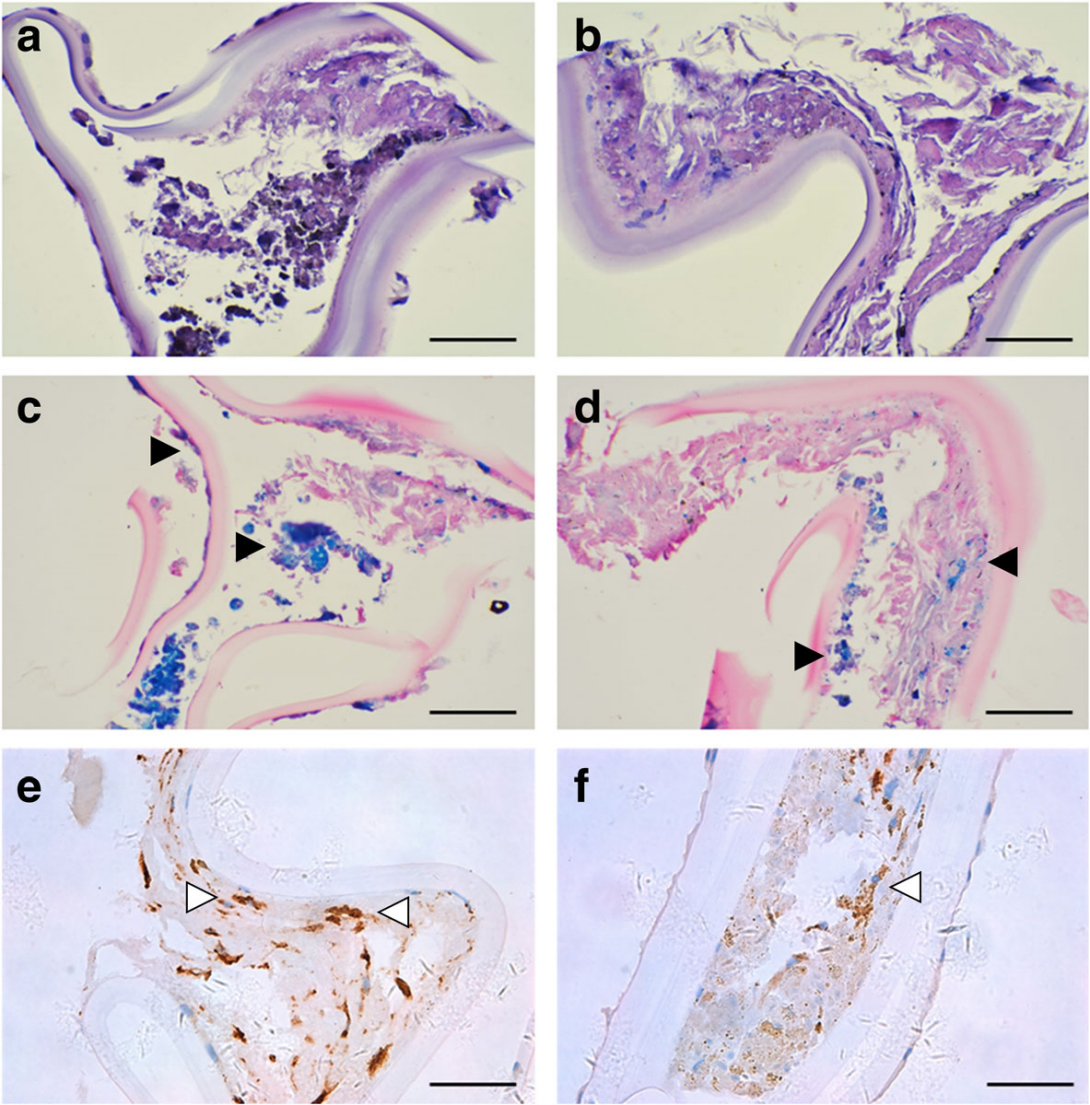
Histopathologic results for the anterior lens capsule with ocular siderosis. **a**, **b** Hematoxylin and eosinstaining showed scattered hemosiderin deposits in the anterior capsule. **c**, **d** Prussian blue staining confirmed the presence of iron pigments in the anterior capsule (black triangle). **e**, **f** Cells in the anterior capsule stained strongly for CD18, a classic macrophage marker (white triangle)

Histopathologic examination using hematoxylin and eosinstain and special stains like Prussian blue and CD18, a classic macrophage marker, were also conducted. Hematoxylin and eosin staining showed scattered hemosiderin deposits in the anterior capsule (Fig. 5a and b). Prussian blue staining confirmed the presence of iron pigments in the anterior capsule (Fig. 5c and d). The cells in the anterior capsule were strongly stained for macrophages, as indicated in Fig. 5e and f.

**Fig. 6:**
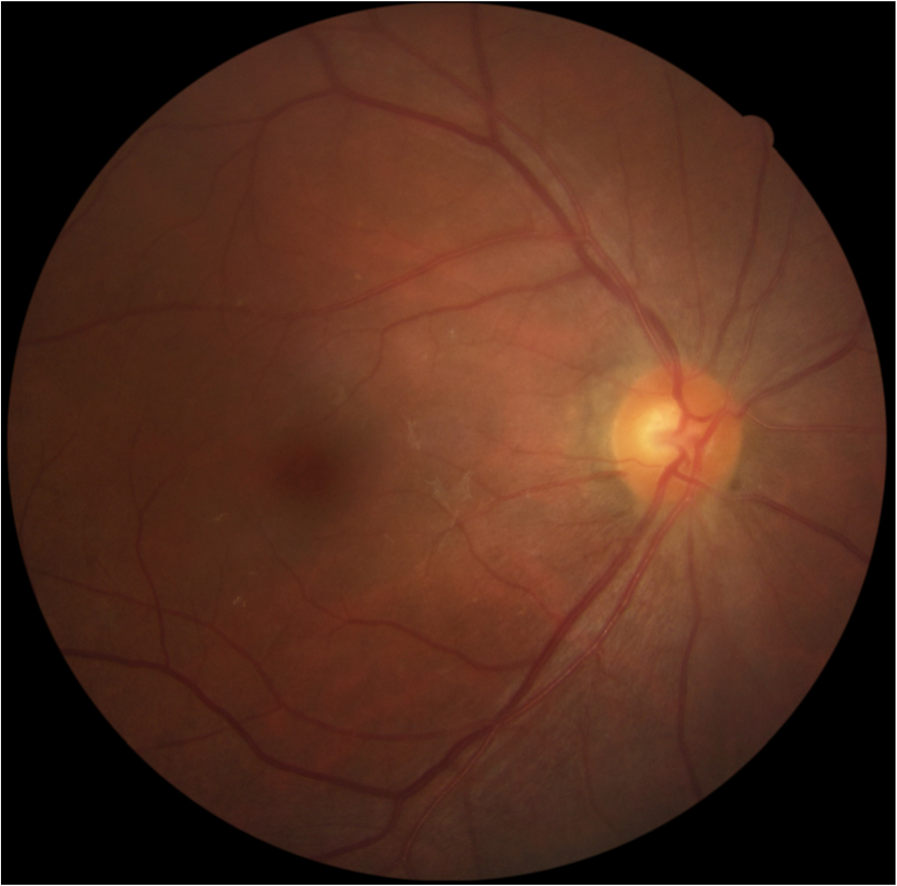
Posterior segment fundus image after the surgery

**Fig. 7:**
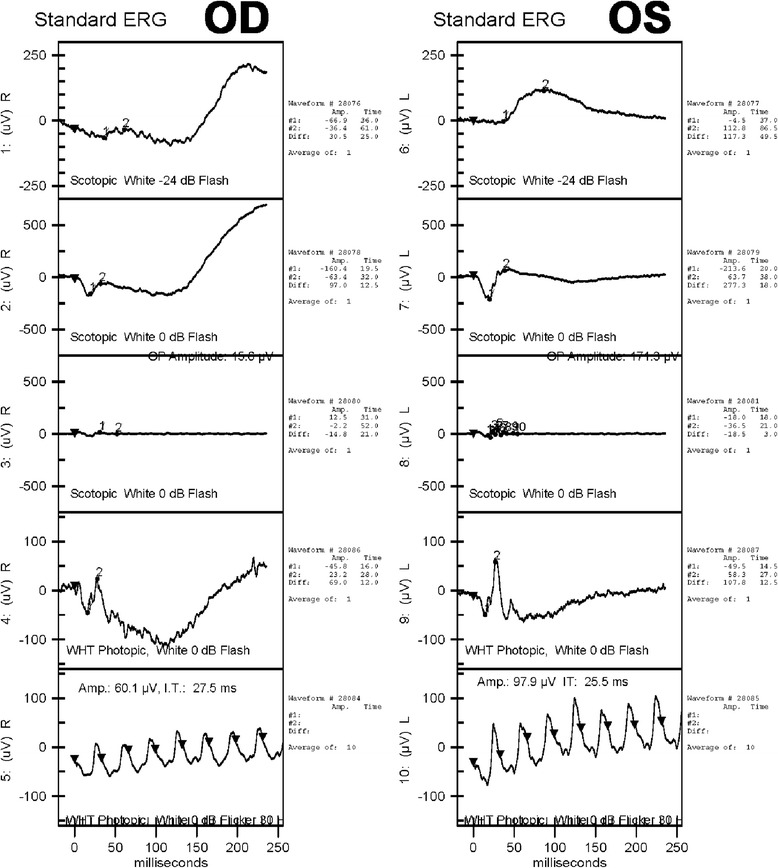
Follow-up electrophysiology test undertaken 6 months after the surgery. (From top to bottom: rod, mixed cone-rod, OP, single-flash cone, and 30-Hz flicker ERG responses)

**Fig. 8:**
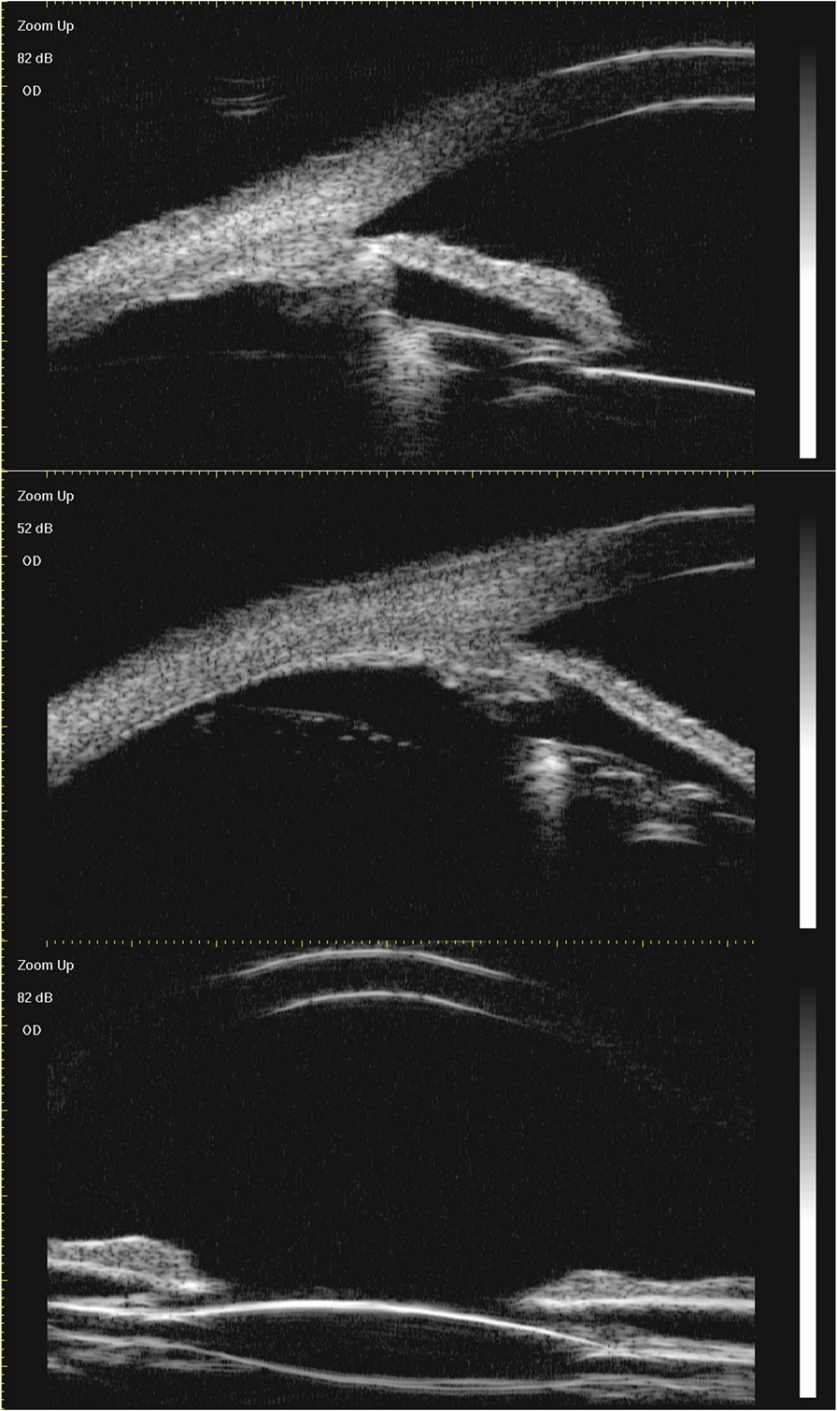
Ultrasound biomicroscopy undertaken at 6 months after the surgery

During the first 3 months of follow-up at our medical center, the patient was satisfied with the surgical outcomes as she obtained a best uncorrected visual acuity of 12/20 and a best corrected visual acuity of 20/20, with an IOL in situ and a stable retina. Six months after the cataract surgery, her best uncorrected visual acuity was still 12/20 and best corrected visual acuity was 20/20. As computed tomography scan cannot detect this tiny foreign body in our case, we conducted serial follow-up examinations 6 months after surgery, including electrophysiology tests, ultrasound biomicroscopy and detailed fundus examination with a three-mirror lens, to check the presence of any intraocular foreign body in this patient. According to the follow-up tests (Figs. 6, 7, and 8), there was no evidence of intraocular foreign body during 6-month postoperative follow-up. At present we recommended the patient to routinely follow up fundus examination and ERG every 3 months.

## Discussions

This woman presented with a one-year history of progressive blurring of vision and a preceding uncertain history of being struck in the right eye by foreign body made of iron. Slit-lamp examination showed a corneal macula inferiorly and an opacified lens with brownish deposits on the anterior capsule. Although no solid evidence of ocular foreign body was observed on the ultrasound B-scan, an orbital X-ray, or computed tomography scan before cataract surgery, the possibility of ocular siderosis caused by deposition of an iron-containing intraocular foreign body was not ruled out. With the assistance of ERG and histopathologic analysis after cataract surgery, the patient was finally diagnosed with ocular siderosis, albeit with no intraocular foreign body.

Iron is a frequent component of metallic intraocular foreign bodies and may lead to ocular siderosis, which commonly presents as reduced visual acuity. Intralenticular foreign bodies account for about 8%–10% of all intraocular foreign bodies. Cataract formation may be an indicator of early siderosis and has been associated with intralenticular foreign bodies [2, 5, 6]. Although computed tomography is still considered to be the gold standard for detection of an occult foreign body, a small intraocular foreign body may be missed with this technique. There has been a previous report of lens siderosis due to an intraocular foreign body missed on imaging such as computed tomography and ultrasonography but later detected perioperatively [5].

A complete ophthalmic evaluation including imaging studies is essential. The diagnosis of an intraocular foreign body is often made by direct visualization on slit-lamp examination or ophthalmoscopy. In this case, there was no intraocular or intralenticular foreign body detected clinically or on imaging, but the patient still developed lens siderosis. The optic nerve and retina were healthy, so there was no associated afferent papillary defect. However, in the right eye, slit-lamp biomicroscopy revealed a corneal macula inferiorly, which could indicate a possible perforating wound previously. As mentioned in a previous study, the time between ocular trauma and development of ocular siderosis may be related to the severity of the intraocular toxic reaction [7]. This varies depending on the shape and size of the foreign body, its iron content, and the amount of time it remains within the eye [3]. Therefore, we cannot rule out the possibility of a dislodged or resorbed intraocular foreign body. There may have been a lodged intralenticular foreign body immediately after trauma that was resorbed over time.

ERG is the method most commonly used to detect ocular siderosis, and all patients should have this prior to surgical intervention. Iron retinotoxicity leads to dysfunction of all the layers of the retina, and more severe damage occurs in the inner retina than in the outer retina in the later stages of thiscondition [8]. Rod-dominated responses are predominantly affected because they are more susceptible to iron toxicity than the cone system. Eventually, responses are progressively reduced in amplitude to become undetectable [7].Our patient underwent cataract surgery prior to ERG due to a delayed ERG appointment at our medical center. ERG recordings remain the reference follow-up examination in patients with ocular siderosis, especially since after the surgery, as previous reports have illustrated, many small particles can be released at the inner retinal surface, potentially inducing further toxicity [4]. Therefore, due to the invisible retinal changes caused by siderosis, it is of great importance to follow up ERG in specific interval in a siderosis case that failed to identify intraocular foreign body.

If all means failed to identify the foreign body, the innegligible role of endoscopic ciliary process and ciliary body examination should be addressed. Ophthalmic endoscope has two fundamental surgical advantages, including bypassing anterior segment opacities, and visualizing anteriorly positioned structures such as the ciliary bodies and sub-iris space [9]. It has been reported that endoscope can be applied to observe the ciliary body, periphery retina tear or other pathological changes, the intraocular foreign body at or near ciliary body and the whole retina in the vitrectomy. This makes the extraction of intraocular foreign body at or near the ciliary body and the conduct of cyclophotocoagulation more accurate and simple [10].

Similar to previous studies, histopathologic analysis in our case showed brownish depositions in the epithelial cells of the lens capsule, and iron was identified in the lens capsule by Prussian blue staining. It is noteworthy that we initially used a stain specific for macrophages so that we could see the effect of iron on phagocytosis in the anterior capsule with ocular siderosis. Macrophage activity is closely related to the mechanism and prognosis of ocular siderosis. According to previous studies, vision may be excellent in siderosis with ERG amplitudes of up to 50%, and complete reversal is possible following successful removal of the intraocular foreign body in the early stages of the condition and with amplitudes of up to 40% [8, 11]. Over this limit, macrophage activity may be overwhelmed by the amount of iron load, leading to direct cellular toxicity [4].

With regard to the treatment of cataract associated with ocular siderosis, implantation of an IOL with a capsular tension ring has been recommended in some previous studies. In patients who are strongly suspected to have a history of eye trauma, the lens ligament may be more fragile and too loose to maintain the stability of a posterior chamber IOL. A capsular tension ring is appropriate for patients with ocular siderosis.

## Conclusions

This case report underscores the importance of close monitoring of patients with a history of trauma or previous penetrating injury to the eye, even if there is no intraocular foreign body, because they could develop ocular siderosis at a later stage. All primary care physicians and ophthalmologists should be aware of the possibility of a retained intraocular foreign body in a penetrating ocular injury, particularly when there is a history of high-velocity metallic injury [7]. In view of the sight-threatening complications of siderosis, prompt intervention is indicated to preserve visual acuity and prevent progression of siderosis to involve the posterior segment [3].

Therefore, when decreased visual acuity and lens opacity with brownish pigment is noted in these patients following trauma, cataract surgery followed by histopathologic analysis is of substantial value for further diagnosis. ERG prior to cataract surgery and during follow-up remains important in the prognosis of ocular siderosis.

## Additional file

Additional file 1: Figure S1. Optical coherence tomography images of the right eye before and after surgery. Optical coherence tomography vaguely indicated no retinal detachment in the posterior pole region of the patient’s right eye. (TIF 1859 kb)

## Abbreviations

(ERG): Electroretinography
(IOL): Intraocular lens
(OD): Right eye
(OS): Left eye
