# Supplementary figures and images for: Siderotic cataract with no signs of intraocular foreign body
Source: BMC Ophthalmol. 2017 Mar 14;17:26. doi: 10.1186/s12886-017-0424-4 (PMC5348785; doi:10.1186/s12886-017-0424-4)

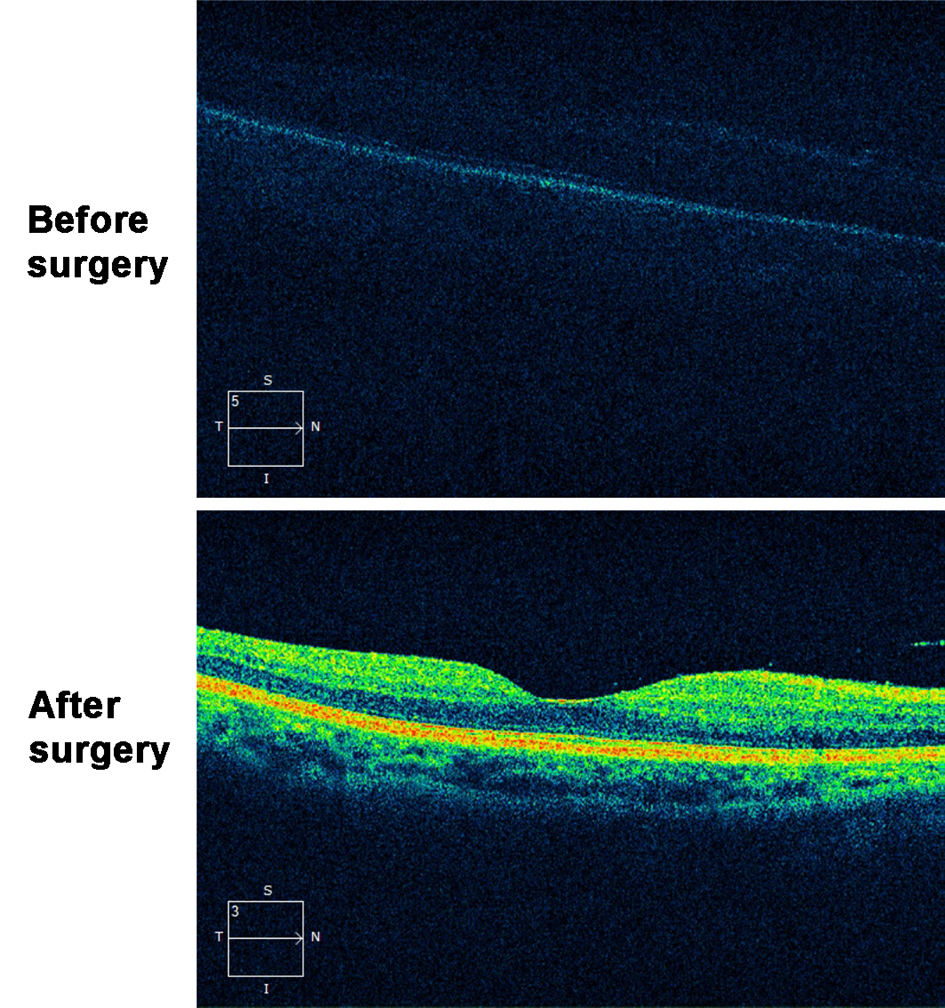

Supplement: Additional file 1: Figure S1. — Optical coherence tomography images of the right eye before and after surgery. Optical coherence tomography vaguely indicated no retinal detachment in the posterior pole region of the patient’s right eye. (TIF 1859 kb) [file 12886_2017_424_MOESM1_ESM.tif]
